# Differentiation of Pancreatic Cancer and Chronic Pancreatitis Using Computer-Aided Diagnosis of Endoscopic Ultrasound (EUS) Images: A Diagnostic Test

**DOI:** 10.1371/journal.pone.0063820

**Published:** 2013-05-21

**Authors:** Maoling Zhu, Can Xu, Jianguo Yu, Yijun Wu, Chunguang Li, Minmin Zhang, Zhendong Jin, Zhaoshen Li

**Affiliations:** 1 Department of Gastroenterology, The First People’s Hospital of Shanghai, Shanghai Jiao Tong University, Shanghai, China; 2 Department of Gastroenterology, Changhai Hospital, Second Military Medical University, Shanghai, China; 3 Department of Electronic Engineering, Fudan University, Shanghai, China; 4 Department of Cardiothoracic Surgery, Changhai Hospital, Second Military Medical University, Shanghai, China; Christian-Albrechts-University Kiel, Germany

## Abstract

**Background:**

Differentiating pancreatic cancer (PC) from normal tissue by computer-aided diagnosis of EUS images were quite useful. The current study was designed to investigate the feasibility of using computer-aided diagnostic (CAD) techniques to extract EUS image parameters for the differential diagnosis of PC and chronic pancreatitis (CP).

**Methodology/Principal Findings:**

This study recruited 262 patients with PC and 126 patients with CP. Typical EUS images were selected from the sample sets. Texture features were extracted from the region of interest using computer-based techniques. Then the distance between class algorithm and sequential forward selection (SFS) algorithm were used for a better combination of features; and, later, a support vector machine (SVM) predictive model was built, trained, and validated. Overall, 105 features of 9 categories were extracted from the EUS images for pattern classification. Of these features, the 16 were selected as a better combination of features. Then, SVM predictive model was built and trained. The total cases were randomly divided into a training set and a testing set. The training set was used to train the SVM, and the testing set was used to evaluate the performance of the SVM. After 200 trials of randomised experiments, the average accuracy, sensitivity, specificity, the positive and negative predictive values of pancreatic cancer were 94.2±0.1749%,96.25±0.4460%, 93.38±0.2076%, 92.21±0.4249% and 96.68±0.1471%, respectively.

**Conclusions/Significance:**

Digital image processing and computer-aided EUS image differentiation technologies are highly accurate and non-invasive. This technology provides a kind of new and valuable diagnostic tool for the clinical determination of PC.

## Introduction

Computer-aided diagnostic (CAD) techniques can assist radiologists to indentify lesions and improve diagnostic accuracy, particularly when used in combination with other physiological and biochemical methods. CAD techniques were used as early as the 1960s [1], and it can help radiologists to detect cancer missed at screening [2]. In 1998, the U.S. Food and Drug Administration (FDA) approved the first CAD system, the Image Checker System from R2 Technology Inc., for use in the early detection of breast cancer. To date, some CAD research findings have been verified by the U.S. FDA; the application of CAD techniques was shown to improve the diagnostic accuracy and reduce the number of misdiagnoses [3]. Based on these successful experience, we previously have implemented the use of digital image processing techniques for the successful differentiation of endoscopic ultrasound (EUS) images depicting pancreatic cancer (PC) from EUS images of non-cancerous samples, including normal samples and samples exhibiting signs of chronic pancreatitis (CP). The diagnostic accuracy reached 98% [4]. These encouraging results indicate that the application of objective, convenient and non-invasive EUS image differentiation systems can significantly improve PC diagnostic procedures.

Early detection and surgical intervention are still the most effective therapeutic methods to improve the survival rate for patients with PC, but, until a late stage, it is notoriously difficult to diagnose [5]. However, the 5-year survival rate of PC patients is below 5% [6], [7]. Although PC and CP are encountered frequently, their clinical differentiation in the early stages remains challenged. Currently, the diagnostic sensitivity of EUS for pancreatic disorders ranges from 85% to 90% [8]–[10], and this technique owned significant advantages compared with other diagnostic methods. However, the EUS-image-based diagnosis is affected by the practitioner’s experience and subjective variables. In particular, EUS-FNA testing and diagnosis depend predominately on accurate EUS image interpretation for the identification of regions of interest; therefore, EUS-FNA tests are known to have very high false negative rates [11]–[13] under some clinical circumstances. Therefore, to understand the value of CAD techniques in the differential diagnosis of PC and CP, this study used a support vector machine (SVM) classifier to test and verify it.

## Results

### Texture Feature Selection

**Figure 1:**
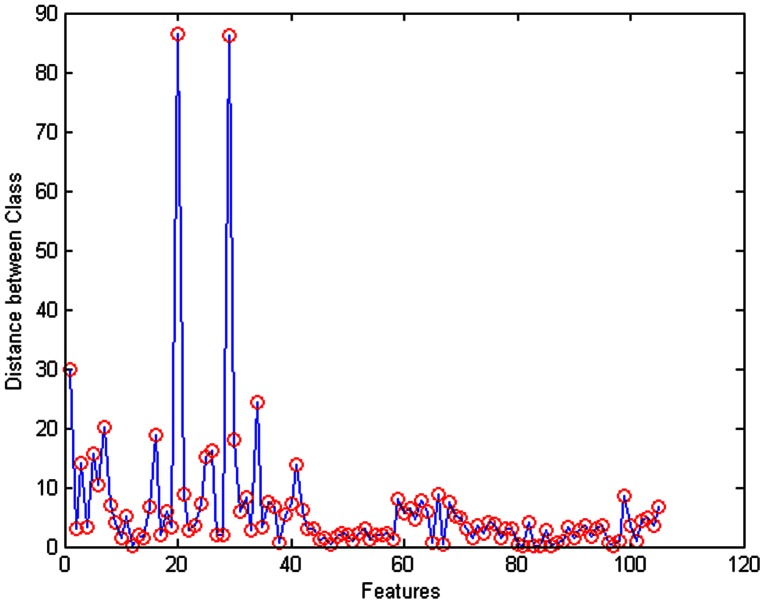
Distance between class algorithm. The vertical axis represents the distance between class, and the horizontal axis represents the corresponding features. A larger distance on the vertical axis indicates better classification results. According to this principle, 25 features are selected to achieve more accurate classification results.

A total of 262 and 126 ROIs in groups of pancreatic cancer and chronic pancreatitis, were available for analysis, respectively. For each ROI, a total of 105 parameters of 9 categories were extracted by the image analysis software in the histogram. Next, we used the distance between class methods to select the 25 better features combination (Figure 1). On the basis of these 25 features, 16 best-classification features of 5 categories was screened to decrease the dimensions of feature vectors and to obtain greater accuracy of classification by using the SFS algorithm. The identified categories and texture features were as follows: 1.grey-level dependence matrix: contrast, invariant moment, entropy, sum of entropy, variance of differences, entropy differences, consistency, absolute value and IMC1; 2. grey level histogram features: standard deviation, consistency and entropy; 3. Shannon entropy of wavelet coefficients: cv2 and cv1; 4. Wavelet coefficients’ standard deviation: ca3; and 5. grey level imaging feature: variance of differences.

### Classification Results

**Figure 2:**
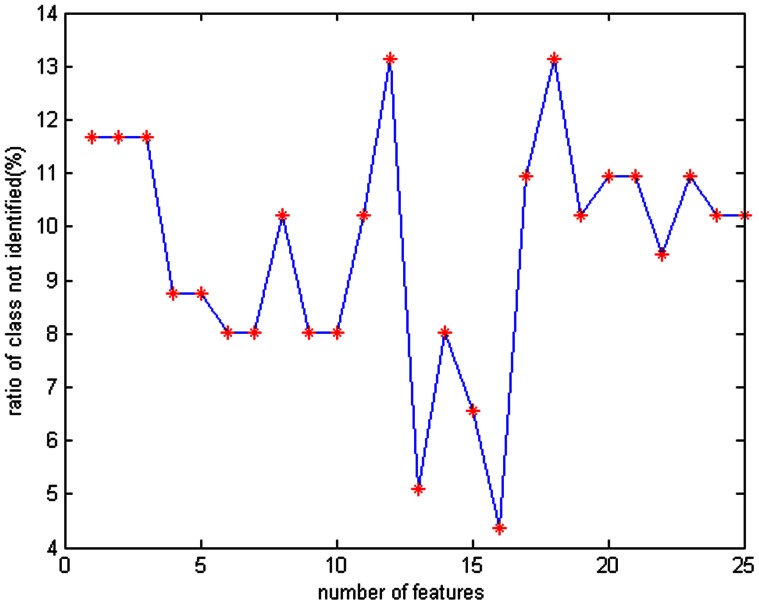
SFS algorithm. The horizontal axis represents the feature, and the vertical axis represents the possibility of inaccurate classification. The texture features identified using the distance between class algorithm were added one by one. The lowest error classification rate was observed when the first 16 features were added.

As the number of cases was limited, an SVM for small sample sizes was used to evaluate the classification performance of image features. All pancreatic EUS images were selected. First, we applied the half-and-half method and the SVM to obtain the correct classification rate (CCR) for evaluating the classification performance of features vectors of different dimensions. In total, 200 random trials were performed to minimise the errors due to the limited sample size. Next, the SFS algorithm was used to add additional texture features one by one from the preliminary selection of 25 features. And a classification error rate as low as 4.38% (Figure 2) (Table 1) was achieved when 16 features were added. Next, the leave-one-out algorithm was applied to further validate the classification performance of the SVM model whose results are presented as the mean. The quantitative results are shown in Table 2.

**Table 1:** A sequential forward selection (SFS) algorithm was used to gain the best combination of features; the correct classification rate (CCR) for SVM was quantitative.

Feature No	1	2	3	4	5	6	7	8	9	10	11	12	13
CCR (%)	88.32	88.32	88.32	91.24	91.24	91.97	91.97	89.78	91.97	91.97	89.78	86.86	94.89
Feature No	14	15	16	17	18	19	20	21	22	23	24	25	
CCR (%)	91.97	93.43	95.62	89.05	86.86	89.78	89.05	89.05	90.51	89.05	89.78	89.78	

We found CCR achieved the highest value when the features were added together to 16.

**Table 2:** The quantitative diagnostic results of the computer-aided differentiation of EUS images for the differential diagnosis of pancreatic cancer and chronic pancreatitis compared with two methods.

Parameters	Half-and-half method results	Leave-one-out method results
Accuracy	93.86±0.17%	94.16%
Sensitivity	92.52±0.75%	91.55%
Specificity	93.03±0.20%	95.07%
PPV	91.75±0.66%	93.67%
NPV	94.39±0.12%	96.98%

## Discussion

Over the years, the diagnosis of PC has been hampered by its anatomical location and the limited number of available examination procedures. With the wide application of endoscopic ultrasonography, EUS and EUS-FNA have become the preferred diagnostic methods for PC [14], [15]; these methods exhibit diagnostic accuracies up to 85%, which are significantly higher than the 50% accuracy obtained with CT exam-based diagnoses [16]. However, based on EUS for early diagnosis of pancreatic cancer, the operator's experience and subjective factors have a greater impact on the results; especially in the presence of chronic pancreatitis cases, the inflammatory status observed in patients with CP can interference PC diagnosis, even experienced endoscopists may produce false negative [12]. In addition, the application of the EUS-FNA diagnostic procedure is limited in community hospitals. Furthermore, even when the EUS-FNA procedure is utilised, the diagnosis might also be affected by the location of the needle insertion and the operators’ experience. Additionally, the possibility of trauma, the heavy workload and the economic burden associated with the EUS-FNA procedure should also be considered.

With CAD, which take into account equally the roles of physicians (subjective aspect) and computers (objective aspect), physicians could use the computer output as a “second opinion” to cover the shortage of radiologists and make the final decisions. Although CAD techniques have been applied for the diagnosis of several diseases in clinical practice, and texture features are helpful for improving tumor diagnosis on sonography[17]–[19], few reports exist regarding their use for pancreatic disorders. For the diagnosis of pancreatic cancer, two reports [2], [12] successfully used SVM and neural network analysis of EUS images to different pancreatic cancer from non-cancer, respectively. In our study, we build a CAD system for pancreas EUS which can be investigated in a quantitative and systematic way via automated texture extraction using an SVM classifier, which has been evaluated as a potential mechanism for the design of a classifier responsible for differentiating between malignant and benign lesions [20] with a good performance in medical diagnostic applications [21]. By comparing this study with Das’ study [22]in the classifiers as we had progressed before (Table 3), we know the SVM system is much more suited to manage classifications problems for limited number of training samples. Zhang MM [4] and Das [22] reported high sensitivity and specificity, however, our results were not as excellent as the other two studies’ (Table 4). Importantly, we should note that texture feature analysis focused on the comparison of histopathological changes and differences, but the other two studies were both include large proportion of normal tissue among the non-cancer patients, and their tissue composition was compared with those from pancreatic cancer patients with larger differences, indicating that the texture nature varied greatly. What is more, we used two methods to verify the SVM classification, and these two results were mutual support (Table 2). So our results were also encouraging and our study indicates the superiority of SVM classification and texture feature extraction.

**Table 3:** Compared Support vector machine with artificial neural network.

SVM	ANN
Global minimum	Local minimum
Small sample sets	Large sample sets
Simple, stable, fast	Complex, unstable, low
Structural risk minimization	Empirical risk minimization
Needs to perform multiclass implementation	Naturally handles multiclass classification
Maps the data sets of input space into a higher dimensional feature space	Depends on the dimensionality of the input space

SVM, support vector machine; ANN, artificial neural network.

**Table 4:** Compared the three studies in results.

Author	NP	CP	PC	classifier	CCR	sensitivity	specificity
Das et al [22]	110	99	110	ANN	–	93.00%	92.00%
Zhang MM et al [4]	20	43	153	SVM	97.98%	94.32%	99.45%
This study	0	126	262	SVM	93.86%	92.52%	93.03%

SVM, support vector machine; ANN, artificial neural network. NP, normal pancreas;

CP, chronic pancreatitis; PC, pancreatic cancer; CCR, correct classification rate.

However, there are several limitations associated with our study. First, we obtained the digital EUS images using enhanced/contrast with fixed-sector endoscopic ultrasonography. Thus, future results may vary if different equipment is utilised. Therefore, our results should be verified by repeating the experiments using other brands of EUS equipment. Second, this study utilised a simple SVM classifier, and comparisons to other commonly used classifiers were not performed. Other classifiers, such as neural network analysis systems and Bayes classifiers et al, should be assessed. Additionally, for the selection of the optimal classifier, the sample size should be increased to evaluate the classification performance more accurately. More importantly, in the current study, this differentiated process was not performed in real time which should be a kind of practical utility, just as most EUS processing modules currently have a built-in capability to perform basic but real-time image processing tasks at the touch of a button.

In summary, this study successfully assessed the ability of EUS image differentiation system to distinguish PC and CP images based on a support vector machine. Overall, the system achieved relatively high classification accuracy. Once a computer-aided EUS image analysis system with real-time diagnosis and auxiliary operation is established, it is very likely that a real-time application can be developed as add-on software. Then, its non-invasiveness, objectivity, simplicity and high efficiency could provide a valuable reference tool for the clinical diagnosis of PC.

## Patients and Methods

### Patients

Our research was a retrospective and single-center design study. We just only analyze correlation between EUS image features and pancreatic diseases. In addition, all patients provided the informed written consent. Our work were permitted and approved by Changhai hospital, Second Military Medical University. A review of the endoscopic database in our institution was performed to identify patients with CP and patients with PC. All PC patients with solid pancreatic lesions were randomly selected from the EUS-FNA database which had been established by a positive cytology. Patients with CP were recruited from the EUS/EUS-FNA database and diagnosed on the basis of their clinical presentation, standard CP Sahai diagnostic criteria [23] and were followed up for more than 2 years. All the databases were collected from May 2002 to August 2011(but the deadline of CP was September 2009).

### EUS Image Selection

All EUS examinations were done by experienced endoscopists who had received Endoscopists certificate from the Chinese Gastroenterological Endoscopic Society, by using an EndoEcho UM 2000 ultrasonic endoscope (Olympus Corporation, Tokyo, Japan) with a probe frequency of 7.5 MHz. The salient findings which included regions of interest (ROIs) were recorded as still images by using the freeze button on the echoendoscope. And all these still images collected from the procedures were saved in the Windows bitmap format (.BMP) for further analysis, which was performed on a standard desktop computer. All images were reviewed by blinded, experienced endoscopists who did not know the pathology results. For the images of PC and CP, the boundary of each ultrasonographically identified lesion was manually delineated and all the pixels within the ROIs were averaged together to form a single signal intensity time-series vector per lesion.

### EUS Image Analysis

**Figure 3:**
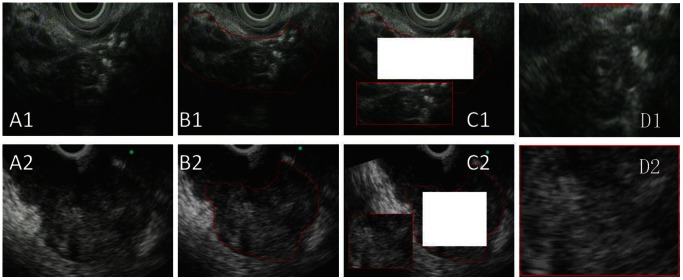
The processes of EUS image selection. As shown in the images of chronic pancreatitis: A1 shows an endoscopic ultrasound image of the head and body of the pancreas. Hyperechoic strands, parenchymal lobularity, hyperechoic foci, many hyperechoic dots with shadowing in the pancreatic parenchyma, and irregular pancreatic duct margins are identified. B1. Delineate the boundary around which contains more chronic pancreatitis features manually with a red circle as a region of interest (ROI). C1. Rectangular sub-images were extracted as large as they could from the ROIs to achieve uniformity of results easily. D1. the histogram was cut from the red circle for extraction of texture features. In the images of pancreatic cancer: A2. Select EUS images with solid pancreatic lesions which had been established by a positive cytology. B2.Delineate the boundary of each ultrasonographically identified lesion manually with a red circle as a region of interest (ROI) around the boundary of visible lesion. C2 and D2 were processed as C1 and D1.

In order to achieve uniformity of results, rectangular sub-images were extracted from the ROIs (Figure 3). These sub-images were analyzed using Matlab R2010a software on a PC Intel Core ™ 2 E8400 3.0 GHz workstation with 3 GB of internal memory. The texture features of each histogram were extracted for the classification of pancreatic EUS images by the image analysis software. However, this procedure actually reduced the discriminatory capacity of the classification function because of the redundancy among different feature vectors. Therefore, further feature selection algorithms were used to reduce the feature vector dimension and improve the classification accuracy. In this study, we used the distance between class and the sequential forward selection (SFS) algorithm for feature selection. The algorithm of the distance between class is a point-by-point process of pixel image classification for a certain image feature that is shared by two classes of images. A greater distance between the median value of the two classes results in a more optimal classification effect. Based on this distance between class algorithm, we first compared the function of extracted features that could be used to differentiate the PC from the CP images. Next, to further compare the performance of different feature vectors, an SFS algorithm was used to identify and select the optimal classification features. Selecting all EUS images of our sample sets and using the leave-1-out algorithm and half-half algorithm respectively in combination with an SVM classifier, the correct classification rate was used to estimate the classification efficiency of features vectors with different dimensions.

### Pattern Classification

An SVM classifier was utilised for pattern classification in this study. The SVM-based classification was implemented by using the libsvm open source library [24].

The SVM is a novel learning algorithm developed from statistical learning theory. The basic idea of SVM classifier is that the EUS imaging as input vectors can be projected into high-dimensional space through pre-defined non-linear mappings. And output two different kinds of vector from the input vector according to the principle of structural risk minimization.

An SVM was used for the classification. We divided the sample database, which comprised 388 cases in total, into a training set and a testing set. The training set was used to train the SVM, and the testing set was used to evaluate the performance of the SVM. Two different methodologies were employed to divide the samples into the training and testing sets. First, a half-and-half method was applied to uniformly divide the sample database into a training set of 194 cases, which included 131 PC cases and 63 CP cases, and a testing set of 194 cases that comprised 131 PC cases and 63 CP cases. In total, 200 trials were performed in order to prevent errors caused by the limited cases. In each trial, the sample database was divided uniformly and randomly to determine the accuracy and standard error of the diagnosis assistance system. Second, a leave-1-out method was applied to evaluate the classification performance. In this method, in each trial, one sample was selected for testing, and the rest of samples were used to train the SVM. This process was then repeated until all the samples were selected for testing.

To evaluate the performance of the experimental results, all data are presented as the mean standard error. The evaluation parameters included the accuracy of average classification (accuracy), sensitivity and specificity. Additionally, positive predictive values (PPV) and negative predictive values (NPV) were calculated.
